# Update on chelating agents in endodontic treatment: A systematic review

**DOI:** 10.4317/jced.60989

**Published:** 2024-04-01

**Authors:** Laura Fortea, Diana Sanz-Serrano, Luciana-Batista Luz, Giulia Bardini, Montse Mercade

**Affiliations:** 1 Department of Dentistry, Universitat de Barcelona, L’Hospitalet de Llobregat, Spain; 2 Department of Dentistry, Universidade Federal do Rio Grande do Sul, Porto Alegre, Brazil; 3 Department of Conservative Dentistry and Endodontics, University of Cagliari, Cagliari, Italy; 4 Researcher at IDIBELL Institute, L’Hospitalet de Llobregat, Spain

## Abstract

**Background:**

The aim of this review was to assess the evidence regarding the most commonly used chelating agents in terms of efficacy, erosive potential, cytotoxicity, interaction, antimicrobial effect, impact on sealers adhesion, and release of growth factors.

**Material and Methods:**

MEDLINE (PubMed) database, Cochrane Library and Scopus were searched up to January 14, 2023, including studies with one or more of the following chelating agents: 17% EDTA, 9% and 18% HEDP, 10% and 20% citric acid, 2%-2.25% peracetic acid and 7% maleic acid. In addition, the reference lists of all selected articles were also checked to identify additional relevant studies. Articles published in English and available in full-text were selected. The quality of studies was assessed using the modified CONSORT checklist guide and the Cochrane Collaboration tool.

**Results:**

The electronic search yielded 538 citations, 56 of which were included. The articles included had moderate and low evidence values. Among 56 articles included, 55 were in vitro studies and one was a randomized clinical trial. Among the in vitro studies, 15 evaluated efficacy and dentin erosion, 12 evaluated interaction with other endodontic irrigants, 9 tested antimicrobial effect, 4 evaluated cytotoxicity in hamster and rat lung cells, 9 evaluated intervention in adhesion of filling materials and 8 focused on release of growth factors and on behavior of stem cells in regenerative endodontic. The RCT tested antimicrobial effect.

**Conclusions:**

17% EDTA is the most effective in smear layer removal and in releasing growth factors on regenerative endodontics. However, the current incorporation of 9% and 18% etidronic acid has shown optimal results due to its compatibility with sodium hypochlorite and its capability on avoiding smear layer formation through a continuous chelation action. Despite these preliminary findings, methodological standardization between studies is required and in vivo studies are necessary to confirm in vitro studies.

** Key words:**Chelating Agents, Smear Layer, Systematic Review, Endodontics, Root Canal Irrigants.

## Introduction

The main objective of root canal treatment is the elimination of bacteria from inside the root canal system. To achieve correct disinfection, it is necessary to use irrigants that are capable of dissolving organic and inorganic matter.

The solutions capable of eliminating the inorganic content are chelating agents, which are acidic substances that remove calcium ions from hydroxyapatite (1). Chelating solutions were introduced in root canal treatment by Nygaard-Ostby in 1957, who recommended the use of a 15% ethylenediaminetetraacetic acid (EDTA) solution with a pH of 7.3 (2). EDTA is a polyaminocarboxylic acid, a strong, colorless, water-soluble chelating agent and has the ability to retain di- and tri-cationic metal ions such as Ca2+ and Fe3+ (2). EDTA solutions are active at pH between 7 and 8 (3). Its usage concentration is 15% to 17% and the time described is based on less than 1 minute if the solution reaches the surface of the root canal wall (2).

EDTA is still the most used chelating agent. However, other substances have also shown excellent results in removing the smear layer (4). Citric acid is a weak organic acid, a strong chelator that reacts with metals to form a soluble nonionic chelate (5). Different usage concentrations ranging from 1 to 50% with a pH of 1 to 2 have been proposed (6).

Another chelating agent used as a final irrigant is peracetic acid (PA). This solution has antibacterial, sporicidal, antifungal and antiviral properties. However, at a concentration of 2-2.25% PA (pH= 2.5), where its effectiveness as a chelator has been seen, it can be caustic when in contact with the oral mucosa and, therefore, its use is recommended. at a lower concentration (7).

Maleic acid is an organic dicarboxylic acid with the ability to remove the smear layer. Ballal *et al*. showed that maleic acid had a similar effect to EDTA in reducing dentin microhardness, but improved roughness (8). Maleic acid is applied at a concentration of 7%, since at higher concentrations the intertubular dentin is affected (8).

An alternative method, called continuous chelation, was introduced in 2005. This involves the simultaneous use of a solution containing NaOCl and a chelating agent during chemical-mechanical preparation (9). Etidronic acid (HEDP-HEBP) has emerged as a viable option, as it can be mixed with sodium hypochlorite without altering its antimicrobial or dissolutive activity in the short term (9). HEBP is a non-nitrogenous bisphosphonate (10) that can also be present in the form of a salt, etidronate, in which the cations are linked to the HEDP anion, usually Na2HEDP or Na4HEDP (11). It is a weak chelating agent that should not be used as a single irrigation and, if used as a final irrigation, requires 300 seconds for optimal results (12). Its use in continuous chelation during biomechanical preparation prevents the formation of the smear layer, in addition to its low toxicity (13,14).

Due to the wide variety of chelating solutions available, this systematic review aims to: (I) update the use of different chelating agents in endodontic treatment (II) based on the results obtained, be able to decide which chelating agent provides adequate characteristics to establish a protocol of use.

## Material and Methods

-Protocol and Registration

The review protocol of this systematic review was registered in PROSPERO International Prospective Register of Systematic Reviews hosted by the National Institute for Health Research, University of York, Centre for Reviews and Dissemination (CRD42023425927). The review was developed based on the Preferred Reporting Items for Systematic reviews and Meta-Analyses (PRISMA) (15,16) (Supplement 1) (http://www.medicinaoral.com/medoralfree01/aop/jced_60989_s01.pdf)

-Research question

The focused question to be addressed was: “During endodontic treatment and pulp regeneration therapies, which chelating agents provide optimal characteristics in terms of efficacy, low erosion and cytotoxicity, no interaction with other irrigants, adhesion of the filling material, high antimicrobial effect and presence of growth factors?

Therefore, in the present study, the PICOS (Patients, Intervention, Comparison, Results, Study Designs) measured were:

P: human teeth, animal models in case of cytotoxicity studies.

I: irrigation with at least one of the chelating agents: 17% EDTA, 9% - 18% HEBP, 10%-20% citric acid, 2%-2.25% peracetic acid and 7% maleic acid.

C: any final irrigant.

O: efficacy, erosion, interaction with other irrigating substances, antimicrobial effect, cytotoxicity, adhesion of filling materials, release of growth factors and stem cell behavior with the use of different chelating agents.

S: preclinical experimental *in vitro* studies and randomized clinical trials.

-Primary outcome was 

The effectiveness and erosion of chelating agents on dentine surface. Efectiveness of chelating agents is their capability to remove the surface film of inorganic (and organic) debris known as smear layer. Due to the chemical nature of the chelating agents, the dentin surface may also be affected, resulting in erosion of the peritubular and intertubular dentin. In vitro evaluation of smear layer removal and erosion is measured scoring images taken by confocal laser scanning microscope and scanning electron microscope.

-The secondary outcome measurements were

Interaction with other irrigants. Because of the acidic nature of chelating irrigants, chemical interactions with sodium hypochlorite can lead to a decrease in its activity calculated as the amount of free available chlorine through iodometric titration, mass spectometry and calcium selective electrode.

Antimicrobial activity: The main antibacterial action during root canal treatment is obtained through the use of sodium hypochlorite, however, chelating substances can decrease the amount of free chlorine available affecting its antimicrobial capacity. In vitro evaluation of antimicrobial activity is measured counting colony-forming units (CFU) and scoring images taken by confocal laser scanning microscope and scanning electron microscope.

Adhesion of filling materials: penetration of sealers on dentinal tubules prevents bacterial leakage and confers greater fracture resistance. Strength is measured by a universal testing machine and the failure load is recorded in Newton.

Cytotoxicity and release of growth factors: during regenerative endodontic treatments, the use of a quelating irrigant allows the release of growth factors embedded in the dentin during dentinogenesis. This irrigants will also be in contact with stem cells afecting its viability, migration and adhesion. Citotoxicity and growth factor release has been evaluated by MTT test, ELISA, SEM, CLSM and cell staining.

-Information sources, search methodology and selection of the reports

A systematic electronic search in the MEDLINE (PubMed) database, Cochrane Library and a manual search in Scopus were independently performed by two reviewers (LS and DS) up to January 14, 2023. Disagreement between the two reviewers was resolved by a team discussion. The search strategy used was as follows: (“Root canal”[Mesh] OR endodontic*) AND (Chelation*[Mesh] OR “Decalcifying agents”) OR (“smear layer”) AND (EDTA OR “Etidronic acid” OR “HEBP”OR“HEDP”OR “Peracetic acid” OR “Citric acid” OR “Maleic acid”) AND ((erosion OR demineralization OR “smear layer removal”) OR (Cytotoxic* OR inflammation*) OR (interaction OR hypochlorite OR chlorhexidine) OR (antimicrobial) OR (“push-out bond” OR “sealer penetration”) OR (“regenerative endodontics” OR “growth factors” OR “stem cells”)).

-Eligibility criteria

The inclusion criteria were:

• Studies published between January 2010 and January 2023.

• Reports including at least one of the following chelating agents with their respective concentration: 17% EDTA, 9% and 18% HEBP, 10% and 20% citric acid, 2%-2.25% peracetic acid and 7% maleic acid. The number of accepted sample per subgroup within a study had to be equal to or greater than five.

• Preclinical In vitro studies with extracted human teeth without caries, mature apex and absence of resorption or endodontic filling that evaluated at least one of the previous chelating agents

• Preclinical experimental studies on cytotoxicity where animal studies were permitted, where at least one of the previous chelating agents was assessed

• RCTs that evaluated at least one of the previous chelating agents

• Reports published in English and available in full-text

• When evaluating the interaction between a chelating agent and NaOCl, the reports should use a NaOCl concentration between 2.5 and 5%.

• The review excluded studies published prior to 2010, studies with a low impact factor (Q3 or Q4) and studies published in non-indexed journals according to Journal Citation Reports™ were also excluded.

-Selection of reports

Two calibrated investigators (LS and DS) removed duplicates and evaluated the records, by using a reference management software (Mendeley). Upon assessing the title and the abstract, the reviewers were calibrated for the inclusion criteria using the first 50 manuscripts obtained from the electronic search. A good level of agreement was reached (k value of 0.87). The potentially interesting articles were selected first by title and then by abstract. All titles and abstracts for which exclusion criteria could not be clearly defined were selected for full-text reading. The full texts of the selected reports were read, and eligibility criteria were applied. Disagreements were resolved a team discussion.

-Data Extraction

Reports satisfying the eligibility criteria were processed for data extraction. Two investigators (LS and DS) were involved in the data extraction, and conflicts were resolved by discussion. Tables were created to summarize the following data (if available): author(s), year of publication, objectives, irrigants used, methodology and results.

-Quality and risk of bias assessment

Two investigators (LS and DS) independently evaluated the quality of the included reports using the Modified CONSORT checklist guide for the preclinical experimental reports (17) and using the Revised Cochrane risk-of-bias tool for randomized trials (RoB 2) for the randomized controlled trial (18). Regarding the Modified CONSORT checklist guide, the mean compliance of all articles was recorded with “yes” or “no”, as well as the percentage of compliance for each parameter (Table 1).

**Table 1:**
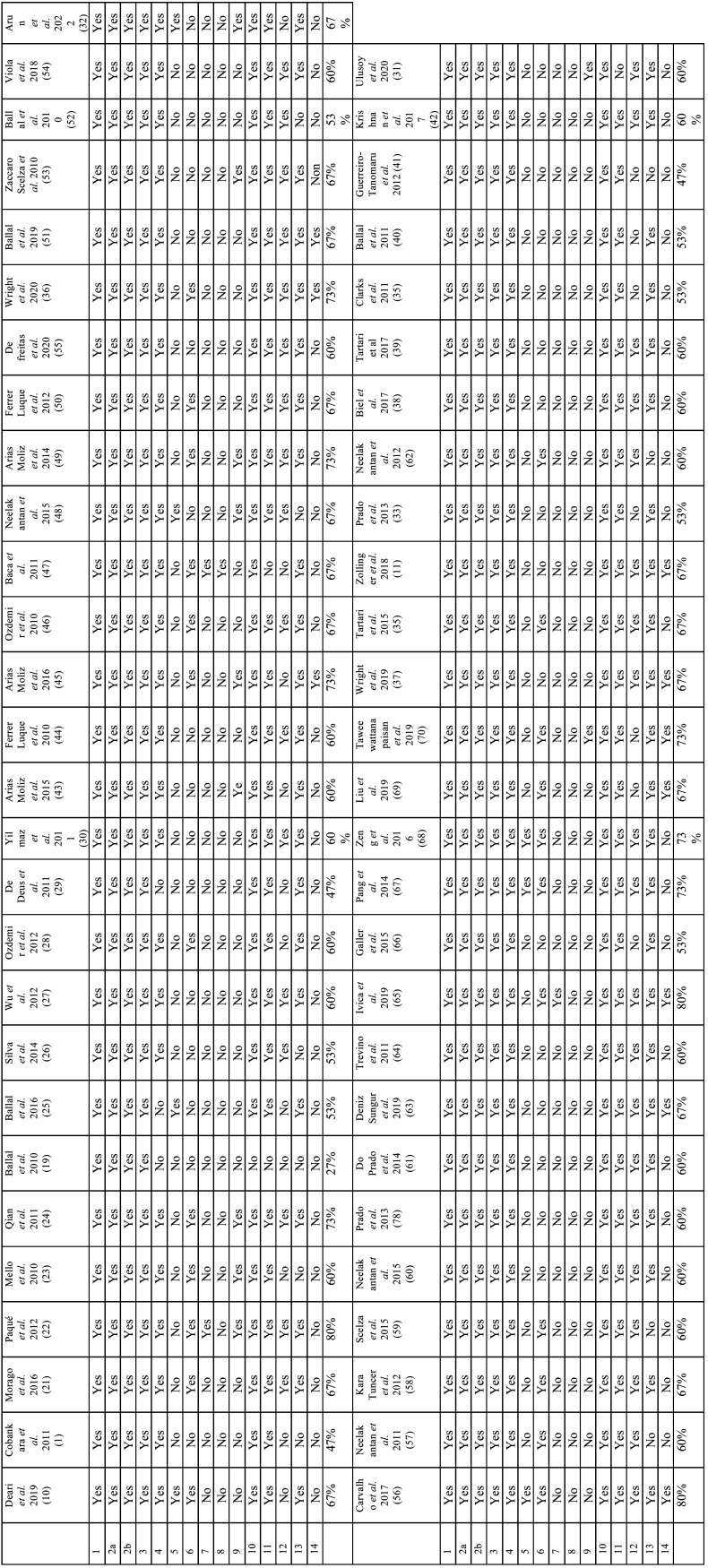
Results of evaluation of in vitro studies using the modified CONSORT checklist.

The publication was grouped into the following categories: low risk of bias if all criteria are fulfill, high risk of bias if one or more criteria are not fulfill, unclear risk of bias when few details were available for classification as ‘high’ or ‘low’ risk.

## Results

-Selection of publications

The electronic search yielded 538 citations, seven reports were manually found as additional eligible articles.

**Figure 1:**
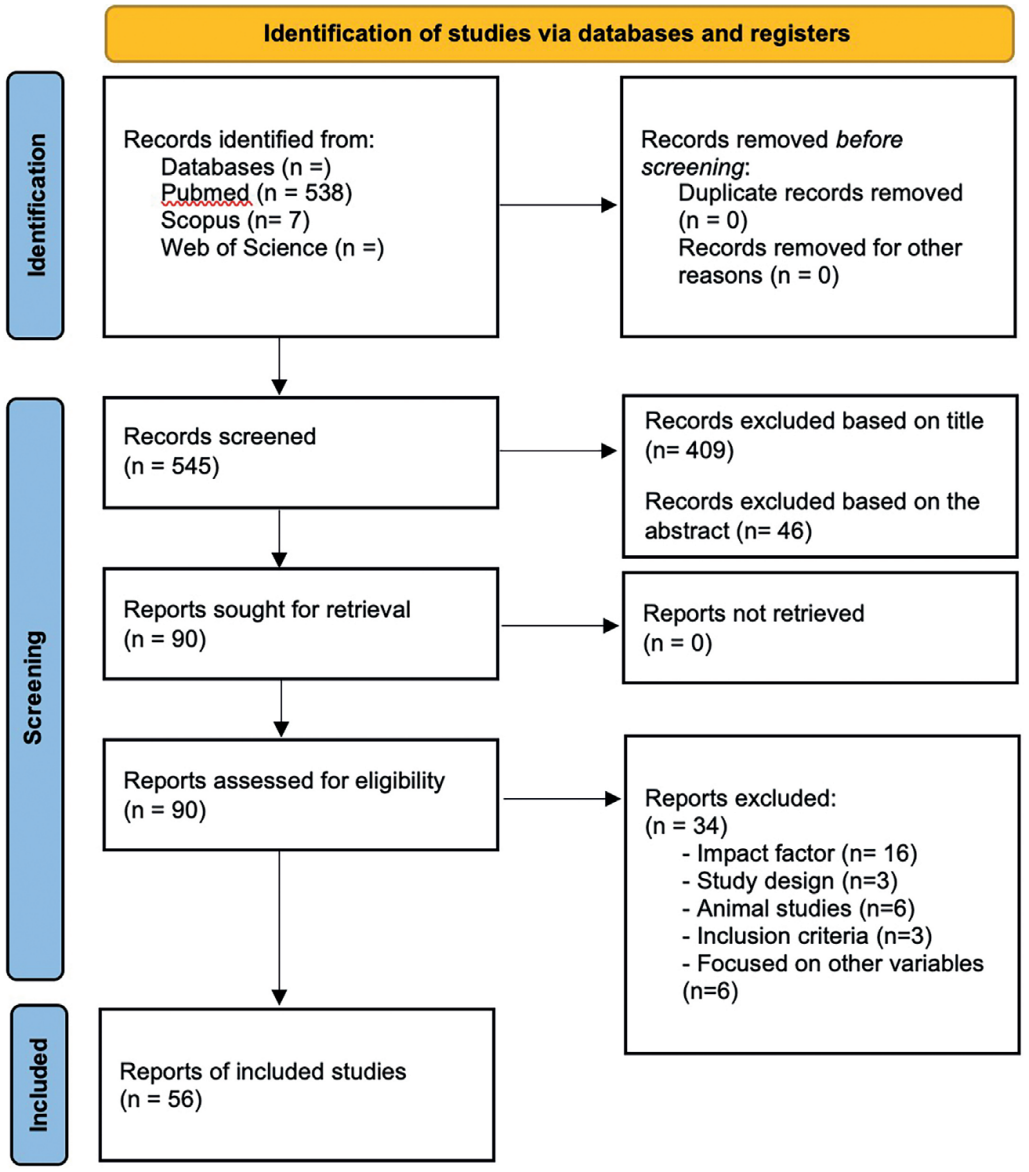
PRISMA flowchart. Selection process for the systematic review according to the PRISMA guidelines.

According to the inclusion criteria, 90 articles were eligible for full-text assessment. After reading the full text, 34 papers were excluded for various reasons (Fig. 1) and 56 articles were included (Fig. 1): 55 *in vitro* studies, and 1 randomized clinical trials were found.

-Quality of the included reports

The Quality of the included *in vitro* studies and RCT are presented in Table 1 and Table 2, respectively. Accordingly to the 55 *in vitro* studies, the average agreement was 62%: all studies obtained a value greater than 47%, except for one that obtained 27% (19). The least observed parameters were those related to blinding and random assignment sequence. On the other hand, those regarding to the structured summary, scientific background and objectives and hypotheses, the intervention for each group, its statistical methods and the results for each group present total agreement (Table 1). The study by Ballal *et al*. (20) was considered “low risk of bias”, as it meets all the conditions set out in ROB2 (Table 2).

**Table 2:**
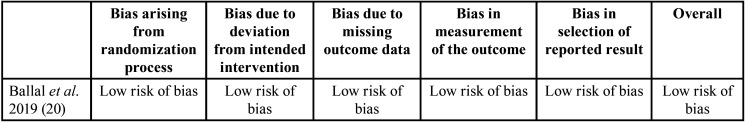
Revised Cochrane risk-of-bias tool for randomized trials (RoB 2).

-Study characteristics

Among the 56 articles on the chelating agents included in this review, 55 were *in vitro* studies and one was a randomized clinical trial. Among the *in vitro* study, 15 evaluated the efficacy and dentin erosion (1,10,19,21-32), 11 evaluated the interaction with other endodontic irrigants (11,33-42), 9 tested the antimicrobial effect (21,43-50), 4 evaluated cytotoxicity in hamster (51,52) and rat (53,54) lung cells, 9 evaluated the intervention in the adhesion of filling materials (33,55-62) and 8 focused on the release of growth factors and on the behavior of stem cells in regenerative endodontics (63-70). The RCT tested the antimicrobial effect (20). The data extracted from the articles selected in this review, as well as the objective, the study design, the samples used, the irrigants with the application time and the main results analyzed are described in Tables  Table 3- Table 8 cont.-1.

**Table 3:**
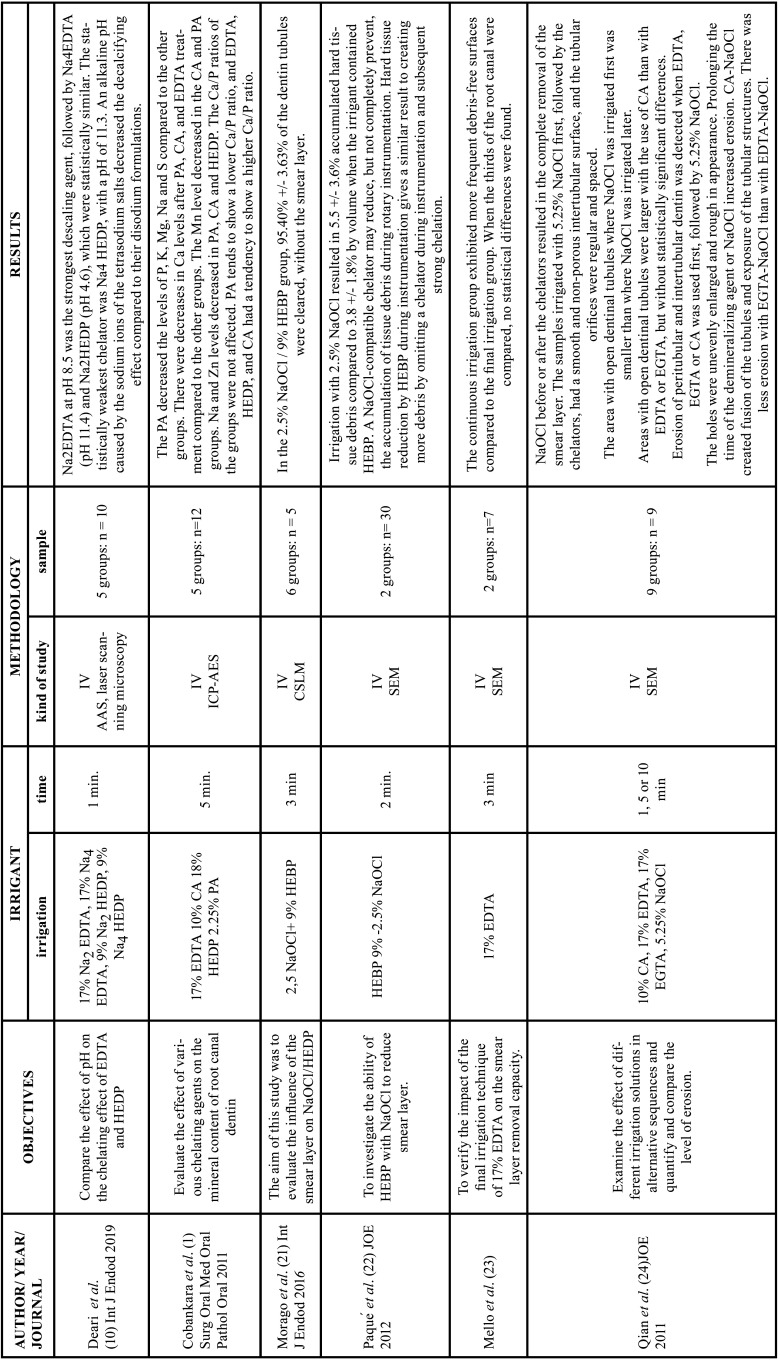
Characteristics of the articles included in the systematic review dealing with erosion and the effectiveness of chelators. EDTA: ethylenediamine tetraacetic acid, CA: citric acid, HEDP: etidronic acid, PA: peracetic acid, MA: maleic acid, NaOCl: sodium hypochlorite, REDTA: 17% EDTA + 0.84 g of cetyltrimethylammonium bromide, EDTA-T: 17% EDTA + 1.25% sodium lauryl sulfate, EGTA: ethylene glycol tetraacetic acid, QMix: 17% EDTA, 2% chlorhexidine and cetyltrimethylammonium bromide, MTAD: Doxycycline, 42% citric acid and detergent, Min: minutes, Sec : seconds, IV: in vitro study, AAS: atomic absorption spectroscopy, ICP-AES: inductively coupled plasma-atomic emission spectrometry (ICP-AES).

**Table 3 cont.**
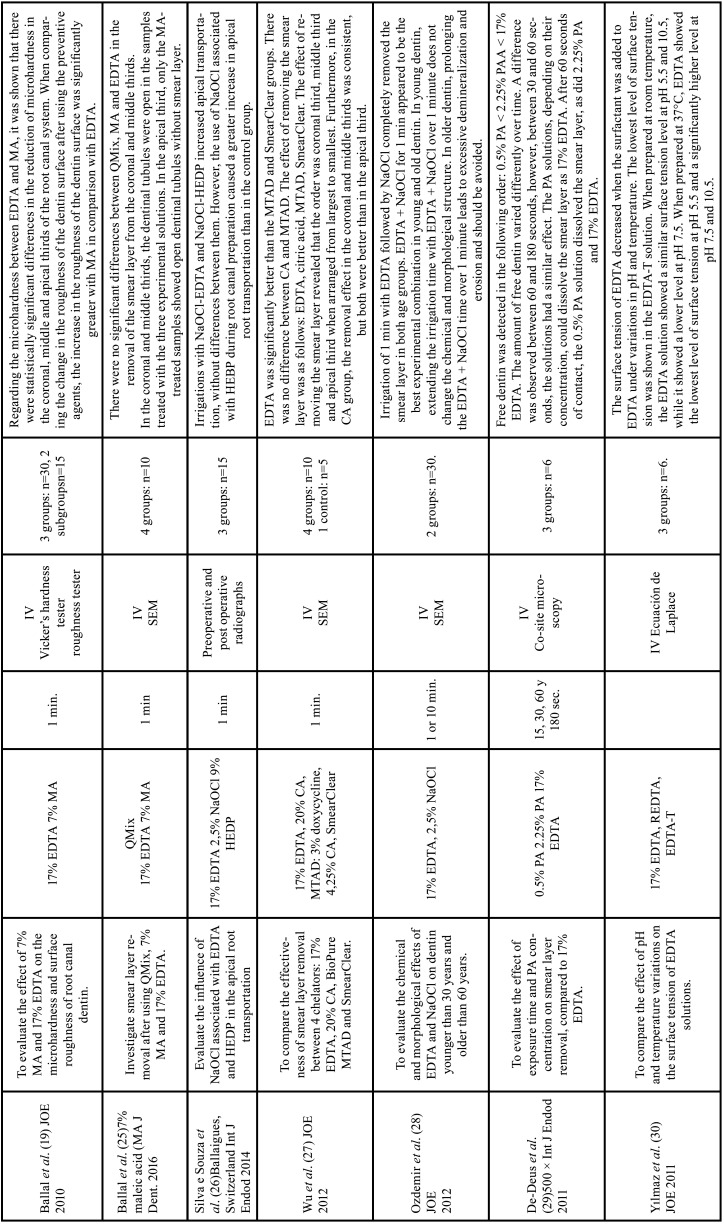
Characteristics of the articles included in the systematic review dealing with erosion and the effectiveness of chelators. EDTA: ethylenediamine tetraacetic acid, CA: citric acid, HEDP: etidronic acid, PA: peracetic acid, MA: maleic acid, NaOCl: sodium hypochlorite, REDTA: 17% EDTA + 0.84 g of cetyltrimethylammonium bromide, EDTA-T: 17% EDTA + 1.25% sodium lauryl sulfate, EGTA: ethylene glycol tetraacetic acid, QMix: 17% EDTA, 2% chlorhexidine and cetyltrimethylammonium bromide, MTAD: Doxycycline, 42% citric acid and detergent, Min: minutes, Sec : seconds, IV: in vitro study, AAS: atomic absorption spectroscopy, ICP-AES: inductively coupled plasma-atomic emission spectrometry (ICP-AES).

**Table 3 cont.-1:**
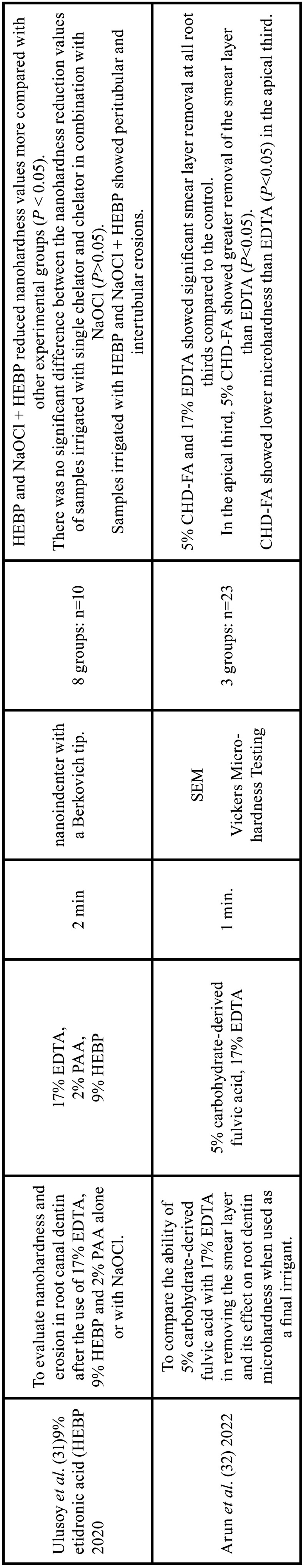
Characteristics of the articles included in the systematic review dealing with erosion and the effectiveness of chelators. EDTA: ethylenediamine tetraacetic acid, CA: citric acid, HEDP: etidronic acid, PA: peracetic acid, MA: maleic acid, NaOCl: sodium hypochlorite, REDTA: 17% EDTA + 0.84 g of cetyltrimethylammonium bromide, EDTA-T: 17% EDTA + 1.25% sodium lauryl sulfate, EGTA: ethylene glycol tetraacetic acid, QMix: 17% EDTA, 2% chlorhexidine and cetyltrimethylammonium bromide, MTAD: Doxycycline, 42% citric acid and detergent, Min: minutes, Sec : seconds, IV: in vitro study, AAS: atomic absorption spectroscopy, ICP-AES: inductively coupled plasma-atomic emission spectrometry (ICP-AES).

**Table 4:**
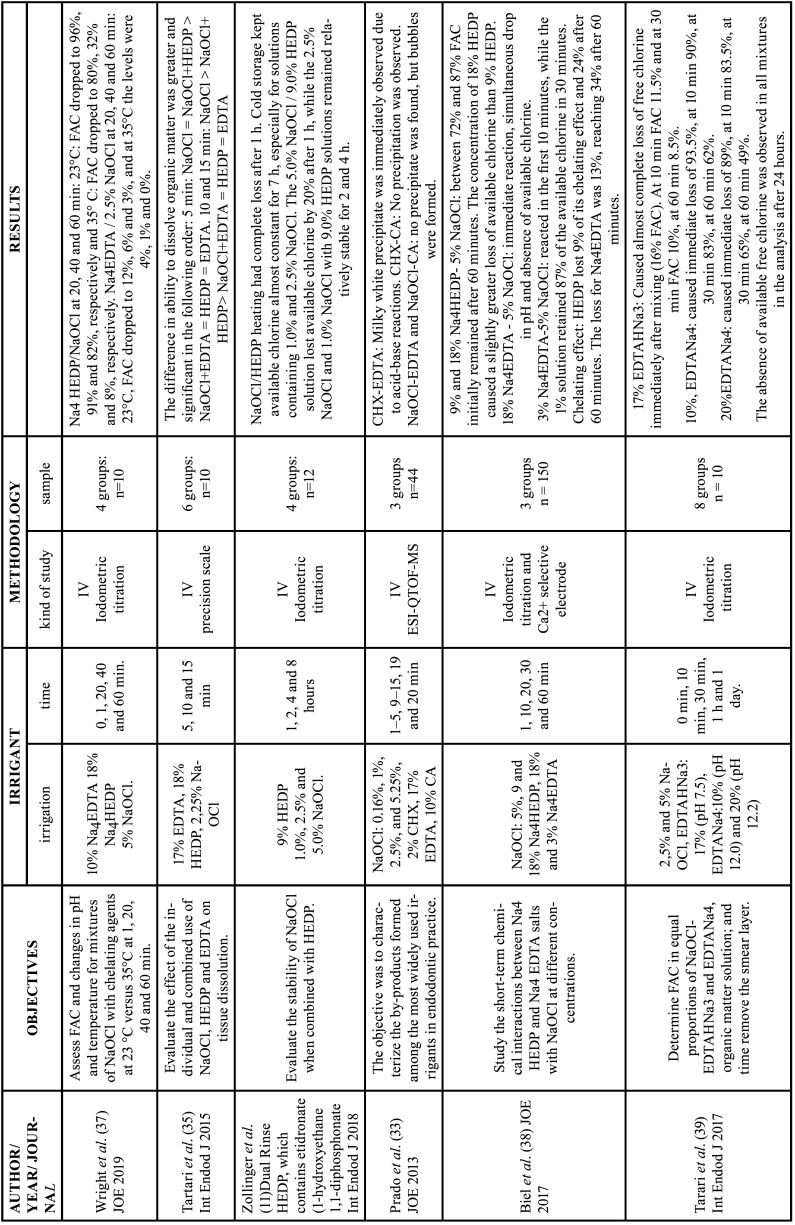
Characteristics of the articles included in the systematic review that deal with the interaction of chelating agents with other irrigating substances. EDTA: ethylenediamine tetraacetic acid, CA: citric acid, HEDP: etidronic acid, PA: peracetic acid, MA: maleic acid, NaOCl: sodium hypochlorite, CHX: chlorhexidine, FAC: free available chlorine, Min: minutes, H: hour, IV: in vitro study, ESI-QTOF-MS: Electrospray Ionization Quadrupole Time-of-Flight Mass Spectrometry, HPLC: High Performance Liquid Chromatography.

**Table 4 cont.**
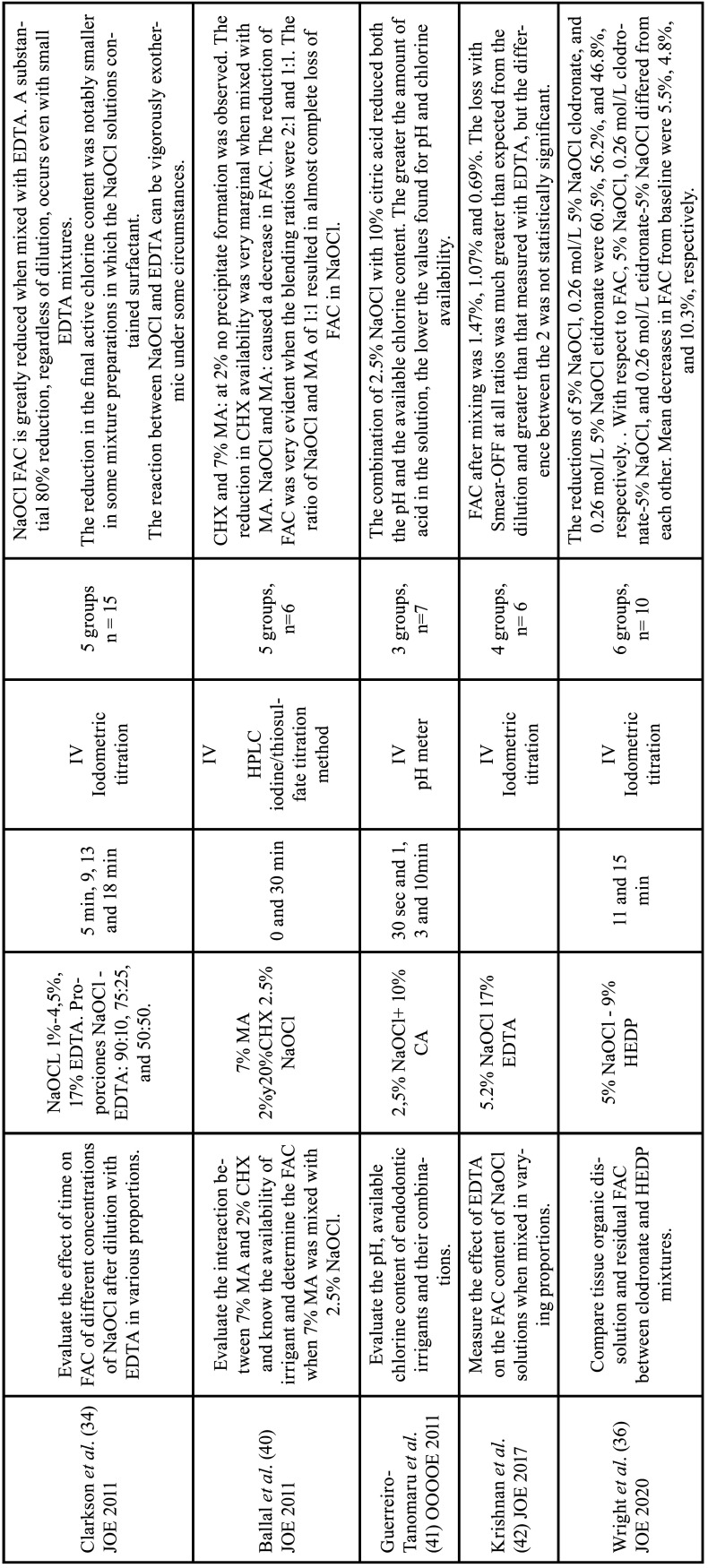
Characteristics of the articles included in the systematic review that deal with the interaction of chelating agents with other irrigating substances. EDTA: ethylenediamine tetraacetic acid, CA: citric acid, HEDP: etidronic acid, PA: peracetic acid, MA: maleic acid, NaOCl: sodium hypochlorite, CHX: chlorhexidine, FAC: free available chlorine, Min: minutes, H: hour, IV: in vitro study, ESI-QTOF-MS: Electrospray Ionization Quadrupole Time-of-Flight Mass Spectrometry, HPLC: High Performance Liquid Chromatography.

**Table 5:**
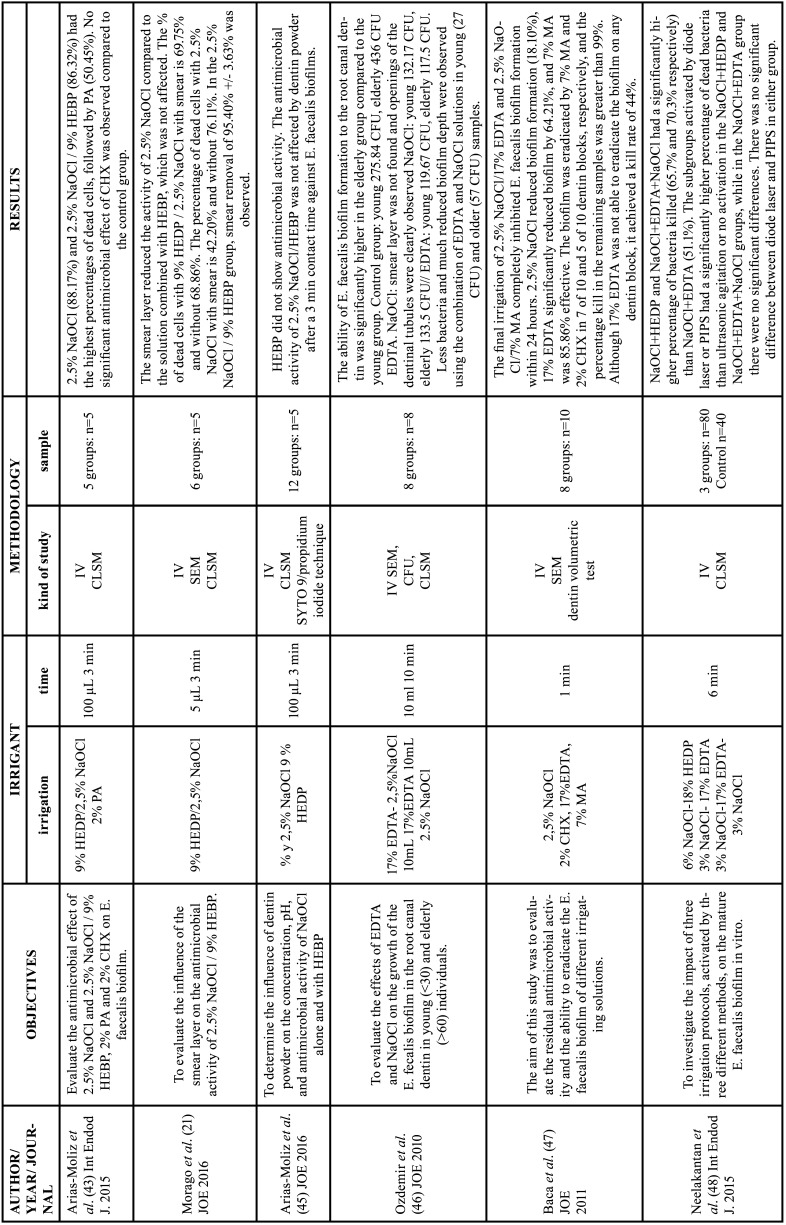
Characteristics of the articles included in the systematic review that deal with the antimicrobial effect of chelators. EDTA: ethylenediamine tetraacetic acid, CA: citric acid, HEDP: etidronic acid, PA: peracetic acid, MA: maleic acid, LA: lactic acid, Hypoclean: NaOCl, cetrimide and polypropylene glycol, Tetraclean: citric acid, cetrimide and polypropylene glycol, NaOCl: sodium hypochlorite, CHX: chlorhexidine, E. faecalis: Enterococcus faecalis, Min: minutes, sec: seconds, IV: in vitro study, CSLM: confocal scanning laser microscopy analysis, SEM: Scanning electron microscopy, CFU: colony forming unit. EDS: energy dispersive spectroscopy FT-IR: Fourier transform infrared spectroscopy.

**Table 6:**
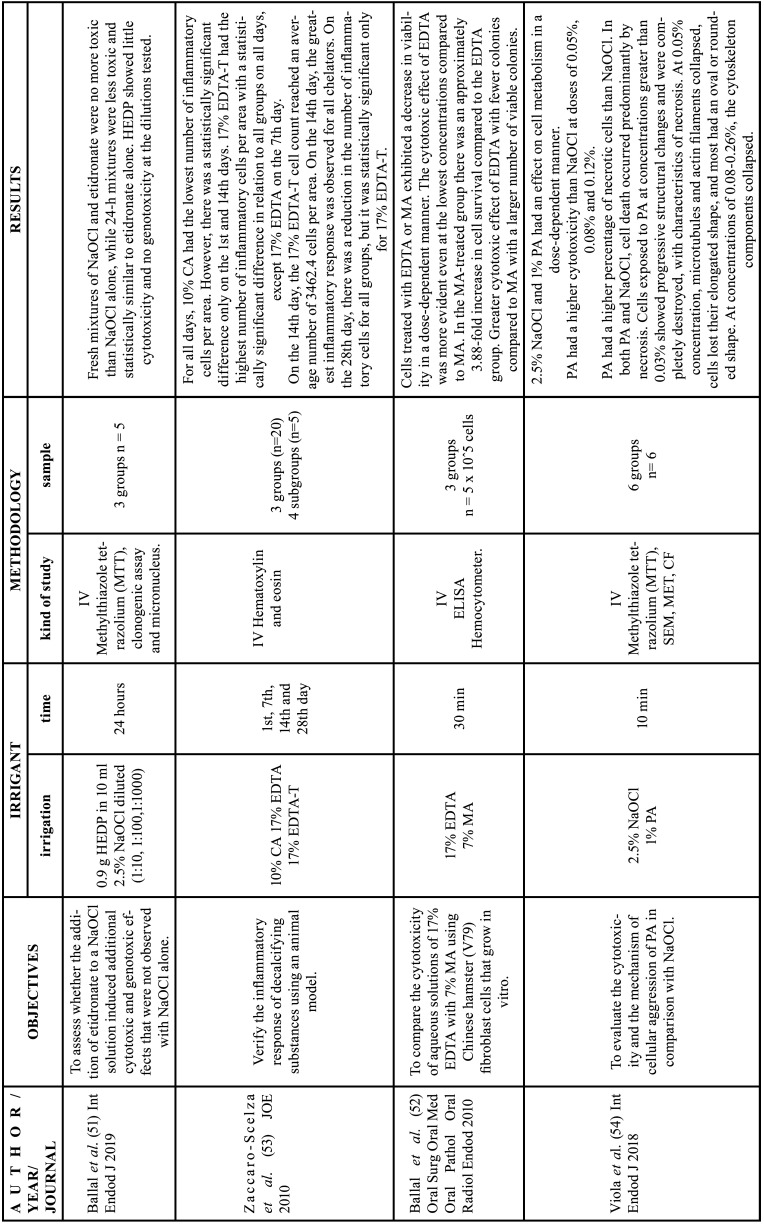
Characteristics of the articles included in the systematic review that deal with the cytotoxicity of chelators. EDTA: ethylenediamine tetraacetic acid, CA: citric acid, HEDP: etidronic acid, PA: peracetic acid, MA: maleic acid, NaOCl: sodium hypochlorite, Min: minutes, IV: in vitro study, CSLM: confocal scanning laser microscopy analysis, SEM: Scanning electron microscopy, TEM: Transmission electron microscopy, CF: Flow cytometry (apoptosis/necrosis), ELISA: Enzyme-linked immunosorbent assay.

**Table 7:**
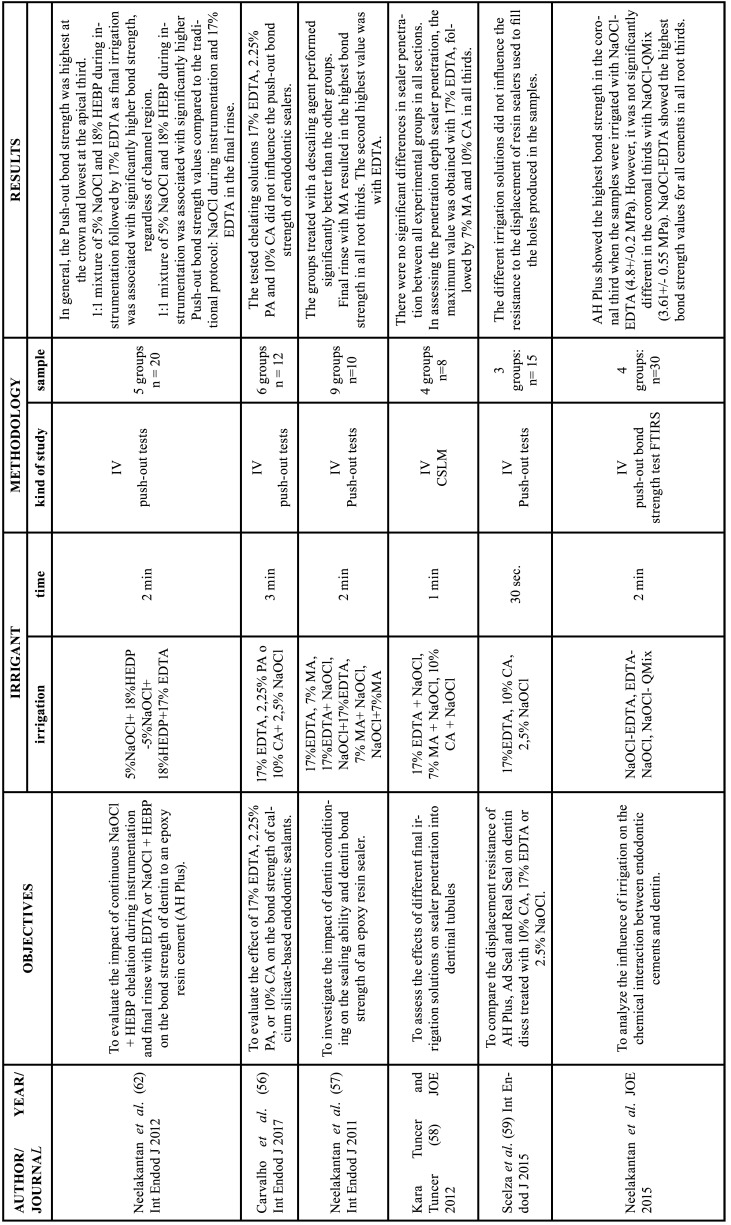
Characteristics of the articles included in the systematic review that deal with the effect of chelators on the adhesion of filling materials.EDTA: ethylenediamine tetraacetic acid, CA: citric acid, HEDP: etidronic acid, PA: peracetic acid, MA: maleic acid, NaOCl: sodium hypochlorite, CHX: chlorhexidine, DW: distilled water, Min: minutes, IV: in vitro study, CSLM: confocal scanning laser microscopy analysis, FTIRS: Fourier transform infrared spectroscopy, AFM: atomic force microscopy.

**Table 7 cont.**
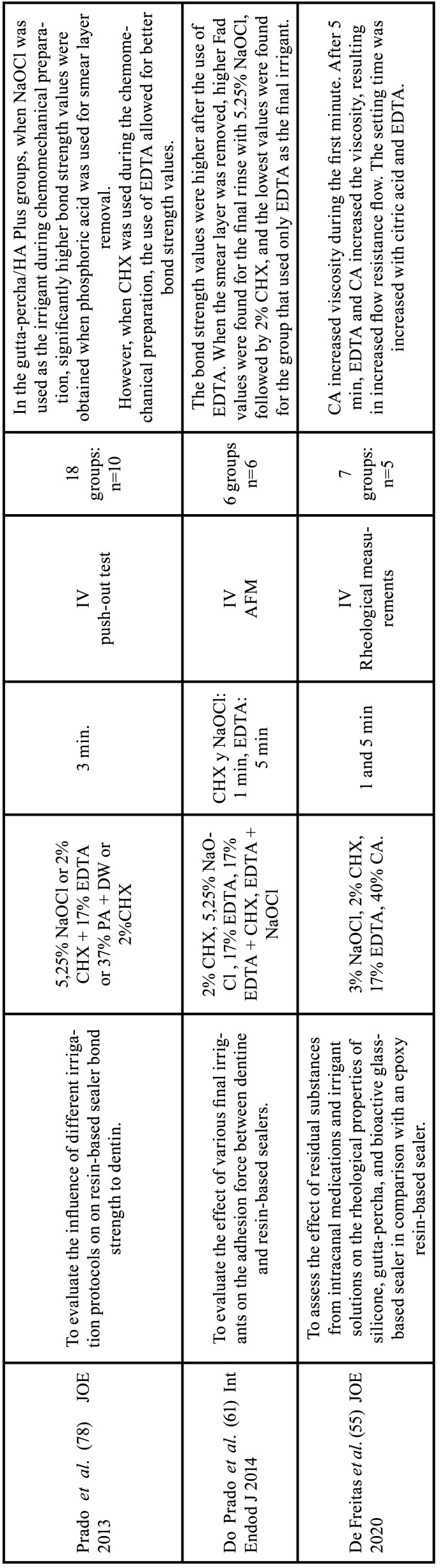
Characteristics of the articles included in the systematic review that deal with the effect of chelators on the adhesion of filling materials.EDTA: ethylenediamine tetraacetic acid, CA: citric acid, HEDP: etidronic acid, PA: peracetic acid, MA: maleic acid, NaOCl: sodium hypochlorite, CHX: chlorhexidine, DW: distilled water, Min: minutes, IV: in vitro study, CSLM: confocal scanning laser microscopy analysis, FTIRS: Fourier transform infrared spectroscopy, AFM: atomic force microscopy.

**Table 8:**
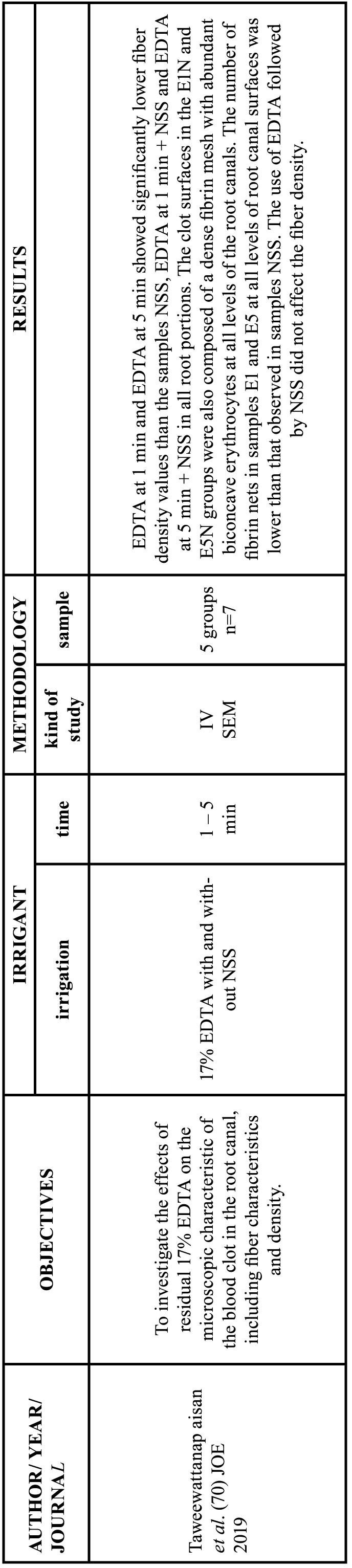
Characteristics of the articles included in the systematic review that deal with the release of growth factors and the behavior of stem cells due to the action of chelators. EDTA: ethylenediamine tetraacetic acid, CA: citric acid, HEDP: etidronic acid, PA: peracetic acid, MA: maleic acid, NaOCl: sodium hypochlorite, CHX: chlorhexidine, DW: distilled water, NSS: normal saline solution, SCAP: Stem Cells From the Apical Papilla. Min: minutes, IV: in vitro study CSLM: confocal scanning laser microscopy analysis, SEM: Scanning electron microscopy, ELISA: Enzyme-linked immunosorbent assay.

**Table 8 cont.**
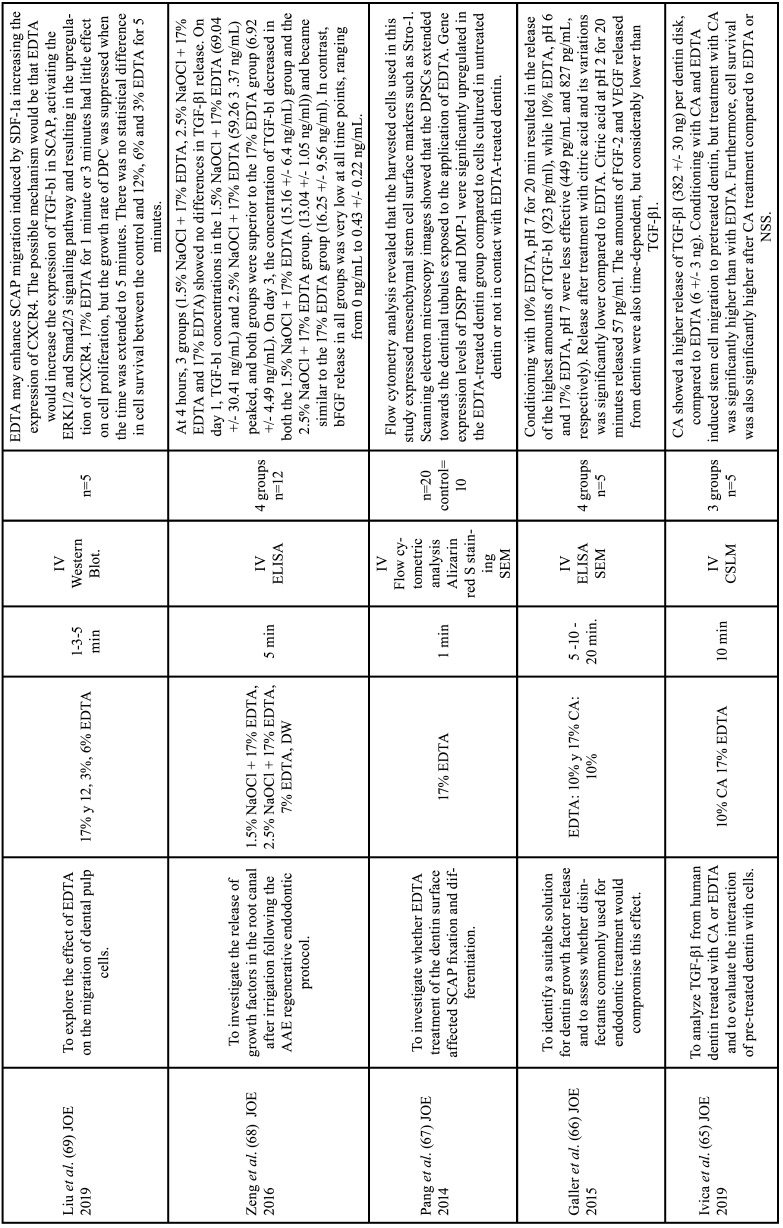
Characteristics of the articles included in the systematic review that deal with the release of growth factors and the behavior of stem cells due to the action of chelators. EDTA: ethylenediamine tetraacetic acid, CA: citric acid, HEDP: etidronic acid, PA: peracetic acid, MA: maleic acid, NaOCl: sodium hypochlorite, CHX: chlorhexidine, DW: distilled water, NSS: normal saline solution, SCAP: Stem Cells From the Apical Papilla. Min: minutes, IV: in vitro study CSLM: confocal scanning laser microscopy analysis, SEM: Scanning electron microscopy, ELISA: Enzyme-linked immunosorbent assay.

**Table 8 cont.-1:**
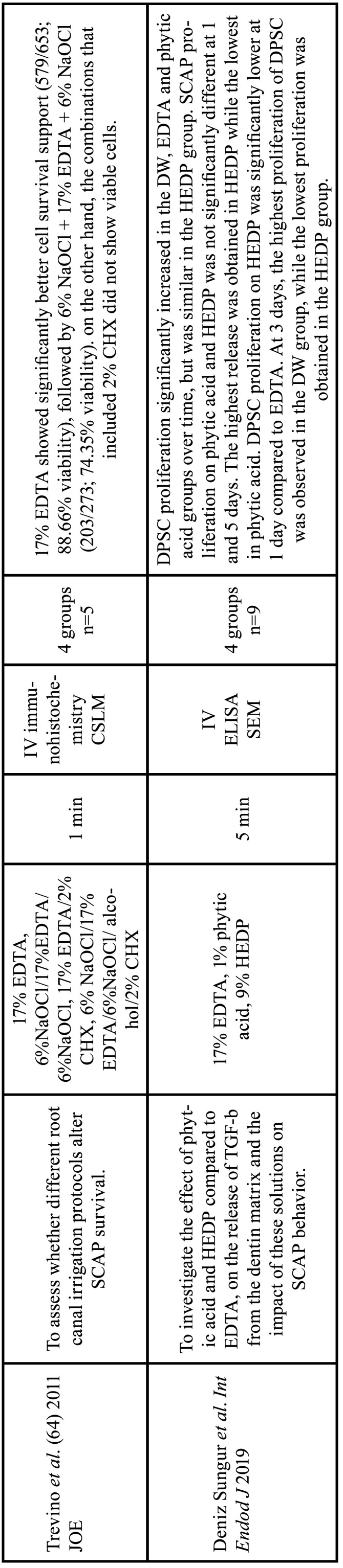
Characteristics of the articles included in the systematic review that deal with the release of growth factors and the behavior of stem cells due to the action of chelators. EDTA: ethylenediamine tetraacetic acid, CA: citric acid, HEDP: etidronic acid, PA: peracetic acid, MA: maleic acid, NaOCl: sodium hypochlorite, CHX: chlorhexidine, DW: distilled water, NSS: normal saline solution, SCAP: Stem Cells From the Apical Papilla. Min: minutes, IV: in vitro study CSLM: confocal scanning laser microscopy analysis, SEM: Scanning electron microscopy, ELISA: Enzyme-linked immunosorbent assay.

-Data extraction

Effectiveness and erosion of chelating agents

The smear layer removal was most effective in the coronal third, followed by the middle third and the apical third (25,27). Regarding the effect of pH and temperature variations on the surface tension of EDTA solutions, Yılmaz *et al*. reported that, at room temperature, the EDTA solution had a similar surface tension level at pH 5.5 and 10.5, while it had a significantly lower level at pH 7.5. At 37°C, EDTA showed a decrease in surface tension at pH 5.5 and a significant increase in tension at pH 7.5 and 10.5. When adding a surfactant to the EDTA solution, the surface tension was greatly reduced, both in pH and temperature variations (30). When evaluating the influence of pH on the chelating effect, Deari *et al*. found that an alkaline pH in the EDTA solution decreased the decalcifying effect of the molecules compared to their disodium formulations (10).

Melo *et al*. observed that continuous irrigation with 17% EDTA had more residue-free surfaces compared to the group in which EDTA was used only in the final irrigation (23). When comparing EDTA with other chelating solutions, Ballal *et al*. found no significant differences between QMix (EDTA, chlorhexidine and a surfactant), 7% maleic acid and 17% EDTA in removing the smear layer from the coronal and middle thirds, showing open dentinal tubules. In the apical third, only samples treated with 7% maleic acid showed open dentinal tubules (25). Wu *et al*. reported that 17% EDTA has a superior ability to remove smear layer compared to 20% CA. MTAD (3% doxycycline, 4.25% citric acid and 0.5% polysorbate 80) showed no difference compared to 20% CA, however, these two solutions were superior to SmearClear (anionic surfactant, cetrimide and 17% EDTA) (27). De Deus *et al*. reported that paracetic acid solutions, depending on their concentration, can be as fast as 17% EDTA in Smear Layer removal. After 60 seconds of contact, 0.5% peracetic acid solution dissolved the smear layer without significant differences with 2.25% peracetic acid and 17% EDTA (29). When comparing the effect of 5% Carbohydrate Derived-Fulvic Acid (CHD-FA) with 17% EDTA, Arun *et al*. (32) reported that CHD-FA was more effective in removing the smear layer in the apical third and presented lower microhardness. Ulusoy *et al*. (31) compared the use of 17% EDTA, 9% HEBP, 2% PAA solutions alone or combined with NaOCl. The authors observed that the HEBP and NaOCl + HEBP solutions presented significantly lower dentin nanohardness values compared to the other irrigants, in addition to presenting peritubular and intertubular erosions.

Three studies evaluated the effectiveness of combining NaOCl with etidronic acid and concluded that continuous irrigation of 2.5% NaOCl with 9% HEDP achieves the same efficacy as chelators commonly used as final irrigants (21,22,26). Four studies evaluated erosion produced by chelating agents (1,19,24,28). Qian *et al*. observed that peritubular and intertubular dentin erosion occurred when the chelating solution was used as the initial irrigant for 1 minute, followed by 5.25% NaOCl. Furthermore, prolonging the time of the chelating agent or NaOCl increased the severity of dentin erosion (24). Ozdemir *et al*. highlighted that in young dentin, prolonging the treatment time with EDTA and NaOCl for 1 minute does not significantly alter the chemical and morphological structure. In contrast, in dentin older than 60 years, prolonging the treatment time with EDTA and NaOCl by 1 minute leads to excessive demineralization (28). Cobankara *et al*. determined that the chelating agent that produces a greater loss of minerals is peracetic acid at a concentration of 2.25%, compared to 17% EDTA, 10% citric acid and 18% etidronic acid (1). Ballal *et al*. showed that there is a greater roughness of the dentin surface in the use of 7% maleic acid compared to 17% EDTA (19).

-Interaction with other endodontic irrigants

Clarkson *et al*. and Krishnan *et al*. reported that when NaOCl was mixed with small amounts of EDTA, NaOCl solutions showed a rapid loss of active chlorine content, showing a linear reduction with time (34,42). Four studies observed that the mixture of NaOCl with HEDP had the same ability to dissolve organic tissue compared to NaOCl alone. In addition, the authors reported that increasing the temperature to 35 °C reduces the therapeutic window from 60 minutes to 20 minutes (11,35-37).

Considering that at alkaline pH, NaOCl does not lose its effectiveness, some authors proposed the use of tetrasodium EDTA (Na4EDTA) (pH12) (37-39). Tartari *et al*. observed that the chlorine availability of 5% NaOCl with 10% EDTANa4 is significantly greater than that of 17% EDTANa3, and the availability of chlorine decreases with time (39). However, Biel *et al*. reported that 5% NaOCl in contact with 18% Na4EDTA showed a decrease in pH immediately, whereas mixing 5% NaOCl and 3% Na4EDTA took 10 minutes to decrease the pH (38).

Two articles evaluated the combination of NaOCl with 7% maleic acid (40) or with 10% citric acid (41), obtaining a decrease in the amount of available chlorine in all the studies (40,41).

When evaluating HEDP combined with 5% NaOCl, Biel *et al*. (38) reported that initially HEDP showed a 9% loss of its chelating effect, and 24% after 60 minutes. The authors also evaluated Na4EDTA combined with 5% NaOCl and the loss of chelating effect was 13% initially and 34% after 60 minutes. However, Na4EDTA and HEDP maintained their chelation capacity during the study (38).

Regarding the by-products formed by the contact between different irrigants, Prado *et al*. (33) observed a milky white precipitate immediately after contacting 2% CHX and 17% EDTA, however, no parachloroaniline was observed (33). However, these formations were not noticed in the mixture of CHX with 10%CA (33). When associating NaOCl with 17%EDTA or with 10%CA, no precipitates were obtained, only the formation of bubbles by the exothermic reaction was observed, which was more evident in the combination with citric acid than with EDTA (33).

-Antimicrobial effect of chelating solutions

Favorable results were found in the elimination of E. faecalis from the dentinal tubules when HEDP was used in combination with NaOCl, compared to NaOCl alone (20,21,43,45,48). However, when HEDP was used alone it had no significant effect against E. faecalis (49).

17% EDTA showed antimicrobial activity when applied alone, eliminating 44% of bacteria, however, when irrigation was performed with 2.5% NaOCl after EDTA, an increase from 70.3% (48) to 100% (47) was observed. Similar results were found by Ozdemir *et al*. in which 17% EDTA and 2.5% NaOCl showed greater antimicrobial activity compared to EDTA alone (46). Furthermore, the reduction was greater in young dentin compared to sclerotic dentin (46). Regarding residual antimicrobial activity, during 5 days EDTA maintained its active action at up to 80%, and during 60 days, it maintained at up to 20% (50), with an average of 64.12% in its residual inhibitory activity (47).

7% MA showed a capacity to eliminate 99% of the bacterial biofilm (47). Its residual activity over five days remained at 100% (50), but at sixty days it decreased by 26% (50) with an average of 85.86% (47). Ferrer-Luque *et al*. reported that the ability of MA to eliminate E. faecalis bioflime is effective, even at lower concentrations, such as 0.88% after 30 seconds of contact and 0.11% after 2 minutes (44).

Only one article evaluated the antimicrobial effect of 2% paracetic acid (43). Arias-Moliz *et al*. reported that after 3 minutes of irrigation with PA, there was a 50.45% reduction in E. Faecalis biofilm (43).

-Chelating agents cytotoxicity

17% EDTA had a more evident cytotoxic effect than citric acid (53) and maleic acid (52). However, EDTA-T showed greater cellular inflammation than EDTA (53). Viola *et al*. reported that fibroblast cells immersed in 1% PA for 10 minutes had a higher percentage of necrosis than cells immersed in 2.5%NaOCl. Cells exposed to PA at concentrations greater than 0.03% had progressive structural changes and were completely destroyed, also with features of necrosis (54). Ballal *et al*. reported that HEDP showed little cytotoxicity or no genotoxicity (51). Additionally, in all studies, the effects on cellular metabolism were observed to be dependent on the concentration of chelating agents (51-54).

-Action on the adhesion of filling materials

Greater adhesion of filling materials was found in groups in which the smear layer was removed (57,61), with greater penetration in the coronal third with a progressive decrease towards the apical third (60,62). In three studies, the authors found no statistically significant differences in sealer penetration between the experimental groups irrigated with 17% EDTA, 7% maleic acid, 10% citric acid or 2.25% peracetic acid (56,58,59).

However, Neelakantan *et al*. reported that MA obtained better results in bond strength than EDTA (57). In another study, final irrigation of EDTA showed better results compared to irrigation of EDTA followed by NaOCl (60). A 1:1 mixture of 5% NaOCl and 18% HEBP during instrumentation followed by 17% EDTA as the final irrigant showed significantly higher bond strength compared to the protocol (NaOCl during instrumentation and 17% EDTA in final irrigation) (62). Regarding viscosity and setting time, De Freitas *et al*. showed that chelating agents increased both the viscosity and the setting time of sealing cements (55).

-Intervention in regenerative endodontic procedures

17% EDTA promoted the migration of stem cells from the apical papilla after a five-minute irrigation, which favored their attachment to the dentinal wall by increasing the expression of TGF-b1 (64,67,69). One study showed that irrigation with NaOCl + 17% EDTA produced more TGF-b1 release compared to irrigation with 17% EDTA alone, with better results at 1.5% NaOCl concentration (68). However, another study reported that the addition of 6% NaOCl to EDTA decreased cell viability compared to EDTA alone (64). Galler *et al*. showed that conditioning of 10% EDTA at pH 7 for twenty minutes resulted in greater release of TGF-b1 compared to 10% EDTA at pH 6 (66). When evaluating the residual effects of EDTA on the blood clot, one study showed that irrigation of 17% EDTA for 1 and 5 minutes had significantly lower fiber density values than 17% EDTA accompanied by saline in all root thirds (70).

Galler *et al*. concluded that irrigation with CA for 20 minutes provided a lower release of TGF-b1 compared to EDTA (66). However, Ivica *et al*. reported that TGF-b1 release was greater with CA compared with EDTA (65). Deniz *et al*. reported greater TGF-b1 release with 9% HEDP compared to 17% EDTA. However, apical papilla stem cell proliferation and viability was higher with EDTA than with 9% HEDP (63).

## Discussion

-Effectiveness and erosion of chelating agents

There is clear evidence that 17% EDTA in association with 2.5% NaOCl for 1 minute eliminated the smear layer significantly (24,25,27,28). However, the use of 17% EDTA alone did not appear to be effective in completely eliminating the smear layer, regardless of contact time (24,28), this can be explained by the action of NaOCl in the elimination of organic matter from the smear layer that favors the action of EDTA. It is important to point out that the same authors are in consensus that increasing the exposure time of EDTA, for more than one minute, and the concentration of NaOCl, in more than 2.5%, produces erosion of the dentinal tubules

(24,25,27,28). In contrast, Mello *et al*. observed a greater detachment of the smear layer with irrigation for 3 minutes, highlighting that the time used in this study did not cause significant unwanted changes in the dentin structure (23).

Ozdemir *et al*. reported that sclerotic dentin has less collagen than younger dentin, favoring greater and faster dissolution of peritubular dentin by acids (28). This could explain the excessive tubular erosion in sclerotic dentin samples, with excessive increase in diameter and tubular area (28). In the apical third, chelating agents were less effective than in the coronal third (27), this may be due to the smaller amount of tubules, smaller diameter and less fluid renewal in the apical third than in the coronal thirds (27).

Wu *et al*. observed that EDTA was significantly more effective than MTAD (27) however, when comparing MTAD with 20% citric acid, no significant differences were found (27). This result can be attributed to the presence of polysorbate 80, which decreases surface tension and increases dentin permeability. However, Wu *et al*. and Ylmaz *et al*. added to EDTA a surfactant such as Smear-Clear, REDTA and EDTA-T, and did not obtain better results than EDTA alone in eliminating the smear layer (27,30). This finding could be caused by the decrease in the pH of EDTA when adding a surfactant, decreasing its chelating capacity. Ballal *et al*. reported that 7% maleic acid had a lower ability to eliminate the smear layer than QMix and 17% EDTA, which may be due to the acidic pH of maleic acid (25).

The results found in relation to etidronic acid are contradictory. Cobankara *et al*. reported less loss of mineral content with HEBP use than with 10% citric acid, 17% EDTA, 2.25% peracetic acid (1). Morago *et al*. showed that continuous chelation was able to obtain 95% of the tubules open, allowing better NaOCl penetration (21), one of the main advantages of this technique is that it avoids the accumulation of a smear layer (21). Additionally, the reactivity with NaOCl is lower, producing less consumption of available free chlorine and thus preserving the ability to dissolve organic tissue (35). Silva e Souza *et al*. reported that the use of NaOCl and EDTA compared with the use of NaOCl and HEDP did not present statistically significant differences for the occurrence of apical deviation. However, unlike the group that received irrigation with NaOCl and EDTA, the group that used NaOCl and HEBP showed a greater increase in root apical transportation than the use of saline solution, which was the control group (26). These results can be explained by the fact that the HEBP solution remains longer in contact with the dentin walls during instrumentation and the NaOCl eliminates the protective collagen of the hydroxyapatite, therefore favoring the penetration of the HEBP, causing a decrease in the microhardness of the surface. Furthermore, Ulusoy *et al*. (31) reported that the use of 9% HEBP showed greater reduction in dentin nanohardness compared to 17% EDTA and 2% PAA. The authors believe that this finding can be attributed to a possible greater wettability and penetration capacity of HEBP compared to other irrigants.

Regarding paracetic acid, the concentration of 2.25% showed greater loss of mineral content compared to other chelating agents (1). However, the 0.5% paracetic acid showed the same results in removing the smear layer compared to EDTA, for 60 seconds (29). Therefore, it is convenient to have more studies with lower concentrations of paracetic acid, especially considering that this substance at 0.5% can be effective in eliminating the smear layer and higher concentrations produce greater dentin erosion.

5% CHD-FA showed better removal of the smear layer in the apical third compared to 17% EDTA. The authors believe that this finding can be attributed to the fact that EDTA has higher surface tension and a higher molecular size than the CHD-FA solution, making its wettability difficult (32).

Another important finding is that when NaOCl is used before the chelator, collagen appears to protect the remaining dentin hydroxyapatite (24). However, the subsequent application of NaOCl facilitates the exposure of the inorganic material through the elimination of the organic matrix and, therefore, the demineralizing effect increases (24).

Results are heterogeneous due to different variables such as root canal anatomy, dentin sclerosis, contact with apical regions, pH. In addition to different concentrations, times and volumes of irrigants used by studies (23,28).

-Interaction with other endodontic irrigants

Solutions of 17% EDTA have a neutral or slightly alkaline pH, at this pH, the reaction between NaOCl and EDTA is exothermic (34,35,42) and the dissolution capacity of NaOCl, which is based on its free chlorine, rapidly decreases (40). Therefore, these substances must be used alternately to avoid the inactivation of NaOCl. Some authors recommend drying the root canal with paper points before using another solution or administering large amounts of NaOCl to eliminate the chelator and achieve the desired effect (40,42). Furthermore, the alkalinization of EDTA with a tetrasodium salt shows greater compatibility with NaOCl (39). However, a greater amount of chlorine and Na4EDTA available in the root canal makes the chemical interaction stronger and the reaction rate increases, therefore, the availability remains insufficient (38).

On the other hand, the interaction between 9% HEDP and 2.5% NaOCl maintains a high concentration of available chlorine for one hour, conserving the ability to dissolve organic tissue (11,36–38). This is probably due to the basic pH of the chelator (11.2), because in an alkaline environment, OCl formation occurs more slowly. As the reaction proceeds, acetic acid, phosphoric acid, oxygen and sodium chloride are formed, therefore, the available chlorine is invariably lost (12).

Biel *et al*. concluded that for clinical application HEDP tetrasodium salt seems much more suiTable than the EDTA counterpart (38). Therefore, HEDP is an alternative as a single irrigant in combination with NaOCl, although the effective shelf life of the solution at room temperature is 60 minutes, decreasing with increasing temperature (37).

The associations of NaOCl with 17%EDTA or with 10%CA, do not present precipitates, only the formation of bubbles caused by the exothermic reaction with CA (33). These bubbles were mainly chlorine gas, a toxic product formed from the increase in the concentration of protons with the presence of chloride ions (3). However, the association of 2% CHX and 17% EDTA produces a milky white precipitate, due to neutralization of the cathode (CHX) and anode (EDTA) (33,71).

The amount of free chlorine in the twelve articles was evaluated by iodometric titration, which allowed a good comparison between them. However, this method does not appear to be accurate, as the available chlorine levels under these conditions are generally higher than the chlorine levels found clinically, due to the instability of NaOCl and the neutralizing effect of dentin and organic matter. Therefore, it would be interesting to obtain *in vivo* chlorine levels using other more accurate methods (72).

-Antimicrobial effect of chelating solutions

The antimicrobial effect of EDTA alone (47) is due to its chelating function that facilitates the mechanical elimination of bacteria, or by producing an inhibition of cellular metabolic pathways (73). In young patients, irrigation with NaOCl and EDTA was more effective than in elderly patients (46), due to the fact that in elderly patients a greater amount of bacteria or a greater content of sclerotic dentin were observed, increasing the volume or contact time of irrigating solutions in these cases may be an option for better results (46).

The fact that continuous chelation with 9% HEDP and 2.5% NaOCl is superior in eliminating Enterococcus faecalis, compared to NaOCl alone (20,43,45,48), can be attributed to better penetration of NaOCl into the dentinal tubules due to the removal of the smear layer by HEDP (74). Furthermore, HEDP has a pH of 11.69, a value above the tolerance of E. Faecalis, which favors the elimination of bacteria (75). However, a lower antimicrobial effect was observed when HEDP was in contact with denser E. faecalis biomasses (49). This finding can be explained by the difference in cell density, as at higher density, there will be greater resistance to alkaline stress (75) and greater ability to neutralize alkaline pH (49).

7%MA is the chelating agent that showed the greatest antimicrobial effectiveness when used alone (47,49,50). The antimicrobial activity of MA resides in the fact that it is an organic acid, with a pH of 1.28, which lowers the internal pH of the bacterial cell and alters the permeability of the cell membrane (73).

2% peracetic acid was more effective than 2% CHX, probably because of the oxidizing agents contained in the peroxide (43). However, this efficacy was lower than that of 2.5% NaOCl due to the bacterial resistance to the oxidative stress of peracetic acid, caused by the presence of enzymes such as peroxidases and catalases (76).

It should be noted that only three studies compared more than one chelating agent in terms of antimicrobial activity (43,47,48). In addition to using different evaluation methods, which, in fact, prevents obtaining a more precise conclusion.

-Chelating agents cytotoxicity

EDTA exhibits greater cytotoxicity and inflammatory response compared to 7% maleic acid and 10% citric acid (52,53). On the other hand, peracetic acid showed dose-dependent cell viability, being lower than 2.5% NaOCl (54). Viola *et al*. observed cells with characteristics of necrosis after the use of peracetic acid and NaOCl, however, this effect was observed to a greater extent with the use of peracetic acid. This can be explained by the fact that peracetic acid is a strong oxidizing agent, which leads to a reduction or loss of cellular enzymatic activity, damaging DNA and causing lipid peroxidation of membranes (54). Unlike peracetic acid, the chelating agent that presented the fewest contraindications was 9% etidronic acid. The authors determined that there were no additional hazards in the combination of 9% HEDP with NaOCl (51). It is worth noting the lack of evidence on this topic, as in recent years there have only been four articles that met the optimal conditions for them to be applicable at the clinical level (51-54). Moreover, only two articles compared more than one chelating agent (52,53). And the four articles used dilutions of chelating agents and very different contact times (51-54).

-Action on the adhesion of filling materials

Prado *et al* and Freitas *et al*. concluded that the strength and sealing ability of adhesives is altered when NaOCl is applied after the chelating agent, with a poor interaction between the adhesive and dentin (55,61). This fact can be explained by the elimination of amino groups from dentinal collagen by NaOCl, or by the oxygen resulting from the decomposition of NaOCl, which inhibits the polymerization of the resin (77). Only one study observed that final irrigation with NaOCl after the elimination of the smear layer is associated with highest adhesion force values (61). However, the authors reported that these results may be associated with the characteristics of epoxy resin-based sealer (AH Plus), which has a higher creep capacity and longer setting time, which may increase the mechanical interlock between root dentin and AH Plus (61).

Regardless of the chelating agent used, the greatest sealer penetration occurred in the coronal third, decreasing consecutively until the apical third (45,57,58). The better elimination of smear layer in the coronal thirds, compared to apical thirds, occurs because there is a greater amount of dentinal tubules in the coronal portion, in addition, the diameter of the coronary tubules are larger than the apical ones. However, Neelakantan *et al*. observed uniform adhesion regardless of the root canal region by continuous chelation. The best results were obtained in the mixture of 5% NaOCl + 18% HEBP during instrumentation and final flushing of 17% EDTA or NaOCl + HEDP, compared to the traditional protocol (NaOCl in the instrumentation and 17% EDTA in the final flushing) (71).

There is heterogeneity in the methodology of the studies. Only two studies used gutta percha (56,59). Six studies used sealers without gutta percha (56,57,59-61,67), of these, only two used canal-like holes (56,59). Another factor worth mentioning is the different contact times of the solutions with the samples. The studies used 30 seconds (59), 1 minute (55,58,61), 2 minutes (57,60,62), 3 minutes (56,78), 5 minutes (55,61) of dentin contact with the chelating agents. It is important to note that both the obturation technique and the effectiveness in removing the smayer layer can influence the penetration of the cement into the root canal walls (58).

Among the studies analyzed in this review, there were no results that could conclude which chelating solution provides better exposure of collagen fibers, a necessary factor for bonding with the adhesive (55,56,59-61). Some studies have reported that chelating agents significantly affect setting time, especially for epoxy resins, this is due to their hydrophobic nature, so it is important to eliminate excess moisture in the root canals (55,61).

-Intervention in regenerative endodontic procedures

Most evidence considers 17% EDTA to be the most effective chelating agent in relation to the TGF-b1 release, favoring cell viability and migration, although its use with NaOCl is contraindicated (66-69). The increase in cell migration in dentin can be explained by the fact that 17% EDTA increases the surface wettability of dentin (67), favoring the adhesion of fibronectin, an important adhesion protein, which preferentially adheres to hydrophilic surfaces.

Taweewattanapaisan *et al*. concluded that after using 17% EDTA, it is important to irrigate the root canal with sterile saline to improve blood fibrin clot formation. This is because calcium ions are important in the clotting process and this process could be interrupted by chelation of residual EDTA (70). These findings were corroborated by Trevino *et al*., who observed that after saline irrigation there was a reduction in precipitates and residual bacteria in the root canal (64).

Only one study compared TGF-b release between EDTA and HEDP (63). Although HEDP showed greater release of TGF-b, it had a decrease in cell proliferation and viability (63). Moreover, there is a lack of further studies to corroborate these results and to investigate the possible cause of the decrease in cell proliferation and viability with etidronic acid.

The ability of citric acid to release growth factors was compared with that of EDTA by two studies, which showed opposite results (65,66). This contradiction may be due to the susceptibility of the ELISA method, used in both studies, to acidic conditions, since protonation causes changes in amino acids and binding proteins that may compete for the same binding points with ELISA antibodies and block the signal from the target protein (79). Therefore, the use of citric acid in regenerative endodontics may be underestimated.

In general, there is a methodological diversity of studies. In some, the dentin was pulverized for measurement, maximizing the release of growth factors. Other studies have used the coronal dentin disc model, where growth factors can be released from all surfaces of the discs. This contrasts with the regenerative endodontic clinical scenario, in which only factors released into the root canal space contribute to the regeneration process.

Likewise, the growth factors released should be quantified after irrigation and not during irrigation, as apical bleeding will be induced after elimination of the chelator. This factor is important to be able to accurately compare the effect of chelating agents in regenerative endodontics.

However, despite the lack of unification in the objectives and the methodological heterogeneity, most evidence shows that 17%EDTA is the most favorable chelating agent for the elimination of the smear layer and for the release of growth factors in regenerative endodontics.

Peracetic acid from 2 to 2.5% was discarded due to its great erosivity, so it is recommended to carry out further studies at a lower concentration. There is a limitation in the number of studies on citric acid and maleic acid, making it impossible to obtain conclusions regarding these two substances.

The scientific quality of the studies included in this review was moderate or low, increasing the risk of bias. The RoB2 tool indicated a low risk of bias for the randomized clinical trial (20), however, the tool does not directly address sample size calculation. In the study by Ballal *et al*. (20) there was no adequate estimate of the sample size, therefore the results should be interpreted more carefully. In addition, most studies are *in vitro*, only one study is a randomized clinical trial, impeding the extrapolation of results to clinical situations. Moreover, there is a lack of protocolization, mainly in relation to irrigation time, volume and concentrations of irrigants.

## Conclusions

- Scientific evidence indicates that 17% EDTA is the most effective in smear layer removal and in release of growth factors on regenerative endodontics, although its use with sodium hypochlorite is contraindicated.

- The current incorporation of 9% and 18% etidronic acid has shown optimal results due to its compatibility with sodium hypochlorite and its capability on avoiding smear layer formation through a continuous chelation action, it may be a promising option, although more evidence are necessary to confirm these results and set an application time.

- Results from *in vitro* studies do not necessarily represent the clinical behavior, although they represent a source of information on the characteristics and properties of the irrigating agents, *in vivo* studies are required.

- Methodological standardization between studies is required to obtain significant conclusions.
